# JMM Profile: *Bordetella pertussis* and whooping cough (pertussis): still a significant cause of infant morbidity and mortality, but vaccine-preventable

**DOI:** 10.1099/jmm.0.001442

**Published:** 2021-10-20

**Authors:** Norman K. Fry, Helen Campbell, Gayatri Amirthalingam

**Affiliations:** ^1^​ Immunisation and Countermeasures Division, Public Health England – National Infection Service, London, UK; ^2^​ Vaccine Preventable Bacteria Section, Public Health England – National Infection Service, London, UK

**Keywords:** acellular vaccine, paroxysmal cough, whole-cell vaccine

## Abstract

Whooping cough (pertussis) is a highly contagious respiratory bacterial infection caused by *Bordetella pertussis* and is an important cause of morbidity and mortality worldwide, particularly in infants. *Bordetella parapertussis* can cause a similar, but usually less severe pertussis-like disease. *Bordetella pertussis* has a number of virulence factors including adhesins and toxins which allow the organism to bind to ciliated epithelial cells in the upper respiratory tract and interfere with host clearance mechanisms. Typical symptoms of pertussis include paroxysmal cough with characteristic whoop and vomiting. Severe complications and deaths occur mostly in infants. Laboratory confirmation can be performed by isolation, detection of genomic DNA or specific antibodies. Childhood vaccination is safe, effective and remains the best control method available. Many countries have replaced whole-cell pertussis vaccines (wP) with acellular pertussis vaccines (aP). Waning protection following immunisation with aP is considered to be more rapid than that from wP. Deployed by resource-rich countries to date, maternal immunisation programmes have also demonstrated high efficacy in preventing hospitalisation and death in infants by passive immunisation through transplacental transfer of maternal antibodies.

## Historical perspective

Pertussis was first described in 1578, but it was not until 1906 that the agent was isolated by Jules Bordet and Octave Gengou in Paris, France [1]. Prior to the development and introduction of whole-cell vaccines in the 1940s-1950s, pertussis was a significant cause of infant morbidity and mortality worldwide [2].

## Clinical presentation

**Fig. 1.**
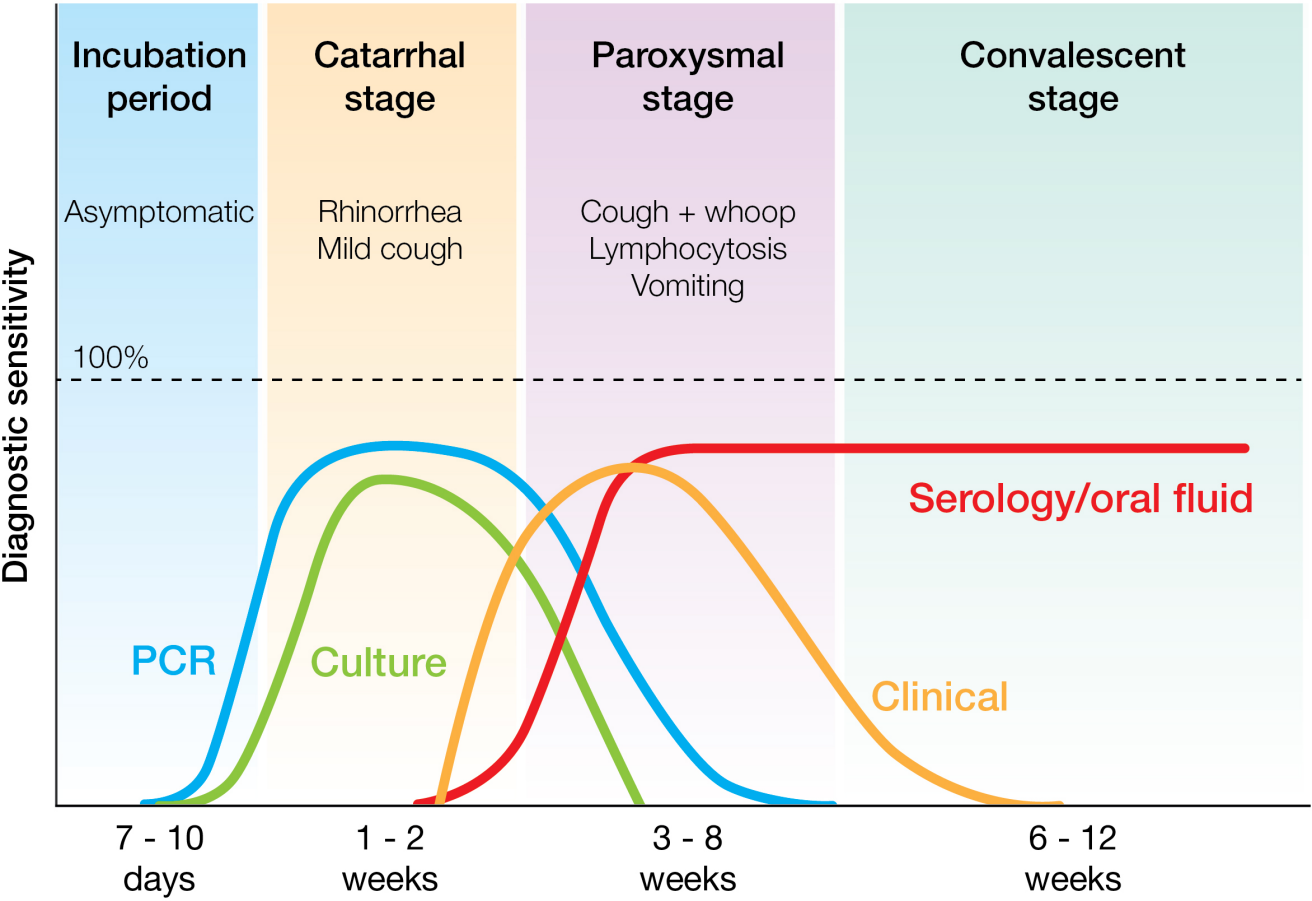
Stages of pertussis infection and relative diagnostic sensitivities of laboratory techniques of culture (green), PCR (blue), serology/oral fluid (red), and clinical diagnosis (orange) during different stages of *Bordetella pertussis* infection.

Typical pertussis infection is divided into (i) asymptomatic incubation period (7–10 days), (ii) catarrhal stage (1–2 weeks) including profuse rhinorrhoea, sneezing, and fever, (iii) paroxysmal stage (1–6 weeks) with paroxysms of intense coughing occasionally followed by a loud whoop and post-tussive vomiting and (iv) convalescent stage (3–4 weeks) with chronic cough (see Fig. 1). Pertussis in infants can require hospitalisation and intensive care, with complications including respiratory problems such as apnoea and pneumonia, together with leucocytosis and pulmonary hypertension [3–5].

## Microbial characteristics: Phenotypic and genotypic features

The genus *Bordetella* is one of ca. 30 genera in the family *Alcaligenaceae *, order *Burkholderiales* within the beta subclass of the *Proteobacteria *. *Bordetella pertussis* is a small, aerobic, non-acid fast, slow-growing Gram-negative coccobacillus with fastidious growth requirements and has no known environmental or animal reservoir. It possesses several adhesins, toxins, and other virulence factors that contribute to disease (see Graphical abstract) some of which are included or targeted in the acellular pertussis vaccines, i.e. pertactin, pertussis toxin (PT) as toxoid, filamentous haemagglutinin (FHA), and the fimbrial proteins Fim2 and Fim3. Many countries have reported emergence of vaccine antigen deficient variants, i.e. *B. pertussis* strains not expressing pertactin and more rarely not expressing PT and FHA [6, 7]. The species shows low genetic diversity by gene content, but long-read whole-genome sequencing technologies have revealed previously unrecognised diversity caused through rearrangements which can affect the expression of virulence factors [8, 9].

*Bordetella pertussis* virulence is characterised by a phenomenon known as phenotypic modulation during which specific genes undergo expression [10, 11]. There are three phases, Bvg+ (virulent), Bvg- (avirulent) and Bvgi (intermediate). However, the signals that cause the phase shift from Bvg+ to Bvgi or Bvg- in the host respiratory tract remain unknown.

## Clinical diagnosis, laboratory confirmation and safety

### Clinical diagnosis

In classic pertussis paroxysms are typically observed consisting of a series of coughs in rapid succession with increasing intensity followed by a large inspiration producing the characteristic ‘whoop’ sound. Diagnosis of atypical/milder pertussis, which can occur in vaccinated individuals, can be difficult, and suspected case definitions include any person in whom a clinician suspects pertussis infection or with an acute cough lasting for ≥14 days, without an apparent cause, plus ≥1 of the following: paroxysms of coughing, post-tussive vomiting or inspiratory whoop. Typical pertussis is more easily diagnosed in infants clinically, whereas if older patients present with cough (only), other causes also require consideration.

### Laboratory confirmation

This is typically achieved by isolation of *B. pertussis *, detection of DNA by PCR, or detection of specific antibodies (see Fig. 1). Recommended specimens for isolation/PCR are nasopharyngeal aspirates, pernasal or nasopharyngeal swabs (or if unavailable, throat swabs), and for estimation of specific antibody levels, serum, or oral fluid. Traditionally Bordet-Gengou agar was used, but increasingly Regan-Lowe (Charcoal blood agar) is now used for isolation. Selective media containing cephalexin is used for primary specimens, and colonies of *B. pertussis* usually appear after incubation for 48–72 h at 35–37 °C. Confirmation of species identity is achieved by phenotypic or molecular methods.

*Bordetella pertussis* PCR assays generally target the insertion element IS*481* due to increased sensitivity from multiple copies present within the genome, but this target is also present in *B. holmesii *, and some *B. bronchiseptica* and thus is often used in combination with other targets [12].

Significant levels of *B. pertussis *-specific antibodies in serum or oral fluid (particularly IgG against PT) can be used to indicate recent infection using enzyme-linked immunosorbent assays (ELISAs) or multiplex immunoassays, but can be confounded by recent vaccination with pertussis-containing vaccines. Each test has its merits, and timing post-onset of cough, antibiotic treatment and vaccination history should be considered [13]. Recommendations for serological diagnosis have been described by European reference laboratories including use of purified PT as coating antigen, thresholds for single serum serology using IgG-anti-PT in International Units per millilitre (IU ml^-1^) and that IgA-anti-PT should only be used with indeterminate IgG-anti-PT levels or when a second sample cannot be obtained [14, 15].

### Laboratory safety

In the United Kingdom, *B. pertussis* is classified in Hazard Group 2 by the Advisory Committee on Dangerous Pathogens (https://www.gov.uk/government/groups/advisory-committee-on-dangerous-pathogens), i.e. can cause human disease, may be a hazard to employees; is unlikely to spread to the community, and there is usually effective prophylaxis or treatment available. Similarly, other organisations classify *B. pertussis* as a Biosafety Level-2 (BSL-2) pathogen or belonging to Risk Group 2. Thus, handling these agents is carried out at BSL-2 using a class I microbiological safety cabinet for potentially aerosol-generating procedures.

## Treatment and resistance

### Treatment

Historically the purpose of chemoprophylaxis was to eradicate *B. pertussis* from cases and prevent secondary transmission. Antibiotics for preventing onward transmission have only demonstrated limited efficacy if given within 7–14 days of onset of illness. Prior to the widespread use of newer macrolides, erythromycin was recommended as the drug of choice, except for infants below 1 month. A Cochrane review of antibiotics for pertussis concluded that although antibiotic therapy for cases was effective in eliminating *B. pertussis* disease to render them non-infectious, it did not alter the subsequent clinical course of the illness [16]. Newer macrolides, e.g. azithromycin and clarithromycin, are now the preferred choice for the treatment and prophylaxis of pertussis, with clarithromycin being the preferred antibiotic for use in neonates due to lower rates of adverse effects.

### Resistance

Macrolide resistance in *B. pertussis* is mediated by A2047G transition in the 23S rRNA gene which prevents the binding of erythromycin and was first reported in the USA in 1994 [17]. Although rare in most countries, a high prevalence of macrolide resistance has been reported in mainland China and it has been hypothesised that this may be linked to the overuse of macrolides in clinical medicine [18–22]. There have also been calls from the pertussis community for wider phenotypic and genotypic testing to improve antimicrobial resistance surveillance data [22].

## Pathogenic strategies: Host range, transmission, infection and virulence factors

### Host range

*Bordetella pertussis* is an exclusively human pathogen with no known natural animal or environmental reservoir.

### Transmission

Pertussis is highly contagious and transmitted from an infected person via respiratory droplets and aerosols. This is usually by coughing or sneezing or when sharing breathing space in close proximity for extended periods of time and in poorly ventilated areas. Infected people are most contagious in the 2 weeks after the onset of cough. In household settings, contacts play an important role in pertussis transmission to infants too young to be protected by primary vaccination [23]. Outbreaks in schools have been reported highlighting the increased risk transmission in such settings [24, 25].

An exciting advance in the understanding of pertussis pathogenesis has been the development of a baboon (*Papio anubis*) model [26]. Using this model, transmission was demonstrated to occur by aerosols and contact. Further, acellular pertussis (aP) vaccination provided protection against disease, but not against colonisation or transmission. This suggests that asymptomatic individuals could transmit pertussis to unprotected infants.

### Infection

*Bordetella pertussis* infects the ciliated epithelium of the airways, and the various virulence factors described (see Graphical abstract and below) contribute to both local and systemic disease pathogenesis in the respiratory tract. Viable organisms can usually recovered from the upper respiratory tract (if appropriate specimens are taken close to onset of cough). Although rare, *B. pertussis* has been isolated from the bloodstream of immunocompromised patients [27].

### Virulence factors

*Bordetella pertussis* infection is due to the interaction of multiple virulence factors with the host, including toxins (pertussis toxin, tracheal cytotoxin, adenylate cyclase toxin and dermonecrotic toxin), adhesins (filamentous haemagglutinin, pertactin, *Bordetella* resistance to killing genetic locus, frame A, tracheal colonization factor), surface antigens, outer membrane proteins and fimbriae. Most virulence factors are activated by a two-component signal transduction system, BvgAS, comprising the sensor kinase BvgS and the response regulator BvgA.

## Epidemiology, prevention and risk groups

### Epidemiology

Pertussis is an important cause of morbidity and mortality worldwide, particularly in infants. Despite current prevention strategies, pertussis continues to be an important public health issue, particularly in developing countries, but also in countries with effective vaccination programmes and high vaccination coverage. Typically, epidemics occur every 3–4 years interspersed between seasonal cycles with a smaller amplitude [2]. Resurgence of pertussis has been reported in several countries despite high vaccination coverage. Proposed reasons for this include: change in diagnostic methods; antigenic variation in the organism, and more rapid waning of immunity after vaccination with aP vaccines. Pertussis is highly infectious, with up to 90% of susceptible household contacts developing the disease and with siblings and parents/guardians important sources of infection for infants.

### Prevention

The primary aim of pertussis vaccination is to reduce the risk of severe pertussis in infants. The development of a whole-cell pertussis vaccine and its widespread introduction into infant immunisation programmes led to a significant reduction in pertussis, and quality-controlled pertussis vaccines (both whole-cell and acellular) have been highly successful in the prevention of severe pertussis in infants.

Pertussis vaccines combined with diphtheria and tetanus toxoids (DTP) have been used in the Expanded Programme on Immunization (EPI) since 1974 [28]. A primary series of three doses of DTP-containing vaccine is recommended, and the first dose may be given as early as 6 weeks of age. A pertussis containing booster dose is recommended for children aged 1–6 years of age (https://www.who.int/immunization/policy/Immunization_routine_table2.pdf).

For countries implementing adult vaccination programmes, the WHO recommends that healthcare workers should be prioritised, especially those with direct contact with pregnant mothers and infants. Vaccination of pregnant women is considered the most cost-effective additional strategy for preventing disease in infants too young to be vaccinated [28, 29].

Transmission can be reduced by good hygiene practices. Universal recommendations are: to wash one’s hands with soap and water where possible (or use alcohol-based solutions); to cover one’s nose and mouth when coughing or sneezing, and to cough/sneeze into a tissue (with proper disposal of contaminated tissues) or into one’s upper sleeve/elbow if unavailable.

### Risk groups

Pertussis can cause severe and life-threatening complications including pneumonia, difficulty in breathing (apnoea) and seizures. Although pertussis can affect all age groups, severe complications and deaths occur mostly in unvaccinated or incompletely vaccinated infants under 6 months.

### Open questions

1. What are the reasons for the resurgence of pertussis in some countries?
2. What are the key features we need to consider in development of novel vaccines?
3. What is the optimal vaccination strategy to protect infants?
4. How can we better integrate epidemiological and laboratory surveillance methodology to improve understanding of pertussis data from different countries?
